# Long-term every-other-day administration of DMAMCL has little effect on aging and age-associated physiological decline in mice

**DOI:** 10.18632/aging.101932

**Published:** 2019-05-02

**Authors:** Zhaomeng Sun, Lijun Zhao, Li Su, Qing Fang, Chenzhong Xu, Yuanyuan Su, Yao Liang, Guodong Li, Yanxue Xue, Tanjun Tong, Jun Chen

**Affiliations:** 1 Peking University Research Center on Aging, Beijing Key Laboratory of Protein Posttranslational Modifications and Cell Function, Department of Biochemistry and Molecular Biology, Department of Integration of Chinese and Western Medicine, School of Basic Medical Science, Peking University, Beijing 100191, China; 2 Center of Medical and Health Analysis, Peking University, Beijing, China; 3 National Institute on Drug Dependence, School of Basic Medical Science, Peking University, Beijing, China

**Keywords:** DMAMCL, NF-κB, inflammaging, age-associated physiological decline

## Abstract

The activation of transcription factor NF-κB is currently identified as one of the driving forces to the aging process. Genetic impairment of NF-κB signaling pathway or pharmacological inhibition of NF-κB activity has been shown to extend healthspan and lifespan in animal models, and delay or reduce many age-related symptoms. However, the aging intervention strategies based on NF-κB inhibition by the suitable small molecular compound is currently still lacking. The water-soluble dimethylaminomicheliolide (DMAMCL), can inhibit NF-κB activity and is currently undergoing clinical trials. In this study, we showed that 15 months of DMAMCL administration started in 1-year old male mice was well-tolerated and safe, and improved or had little effect on some age-associated symptoms, such as neurobehavioral phenotypes, physical performance, cardiac function, hematological parameters, immune aging phenotypes, clinical chemistry parameters, and glucose homeostasis. At the molecular level, DMAMCL administration mitigated serum levels of several age-associated inflammatory cytokines, including IL-6, IL-1α, IL-1β, TNF-α, IFN-γ, and CXCL2, and inhibited NF-κB activity in several aged tissues. Collectively, our results indicate that current strategy of DMAMCL administration may has little effect on aging process in mice, and provide basic clues to further exploit the possibility of DMAMCL-based aging intervention to promote healthy aging.

## Introduction

Aging is characterized by a progressive loss of various physiological functions at the molecular, cellular, tissue, and organismal levels, and ultimately leading to the death of organisms. Aging is a major risk factor for most human chronic diseases, including cancer, diabetes, cardiovascular diseases, osteoporosis, arthritis, and neurodegenerative diseases [1]. Multiple mechanisms are proposed to contribute to the mammalian aging process, such as genomic instability, telomere erosion, epigenetic alterations, mitochondrial dysfunction, nutrient sensing malfunction, inflammation, and etc [2]. Among these, chronic, low-grade, sterile inﬂammation is one of the most important hallmarks in aged organisms, which is termed as ‘inflammaging’ [3]. Mounting evidences show that the blood levels of inﬂammatory cytokines, chemokines, and acute-phase proteins, including interleukin-6 (IL-6), IL-1β, tumor necrosis factor alpha (TNF-α), and high-sensitive C reactive protein (hsCRP), are increased in the older subjects compared to the young subjects, and contribute to many age-related pathologies [4,5].

The transcription factor NF-κB is a master regulator of the cellular response to inflammation [6]. Many studies demonstrate that the NF-κB activity is increased in multiple tissues and organs in old organisms compared to the young organisms [7–9], and in several premature mouse models [10–12]. Importantly, NF-κB is identiﬁed as a major regulator of gene expression programs most strongly associated with mammalian aging in multiple human and mouse tissues [9]. The aging-dependent NF-κB hyperactivation in the hypothalamus is recently also revealed to modulate systemic aging process [13]. Moreover, NF-κB activation has been linked to many age-related diseases, such as atherosclerosis, insulin resistance, neurodegenerative diseases, and cancer, etc [14–21].

Accumulating evidences demonstrate that genetic augment or impairment of NF-κB signaling pathway either accelerate or delay the aging process [22]. Nfkb1-deficient mice display NF-κB hyperactivation and systemic inflammation phenotypes which leads to premature aging and many age-related diseases [23,24], illustrating NF-κB as a bona fide driver of aging process. Genetic activation of NF-κB in hypothalamus also accelerates aging process and shortens lifespan in mice [13]. In Contrast, genetic depletion of NF-κB has demonstrated to extend lifespan in both wild-type and premature mouse models [10–13,25], and attenuate multiple age-related symptoms and pathologies. Similarly, NF-κB inhibition by pharmacological agents, for instance, an 11-amino acid peptide inhibitor of IKK, termed the NEMO-binding domain (8K-NBD), and pyrrolidine dithiocarbamate (PDTC), as well as hypochlorite (HOCl), increases lifespan and reduces age-related pathologies in various animal models [11,26,27]. However, the small molecular compound that can inhibit NF-κB activity and is suitable for long-term anti-aging intervention in mammals is currently still lacking.

Micheliolide (MCL) is a natural guaianolide sesquiterpene lactone (GSL) from *Michelia compressa* and *Michelia champaca* plants [28]. MCL has been reported to suppress dextran sodium sulphate (DSS)-induced inflammatory intestinal disease, colitis-associated cancer, rheumatic arthritis, and LPS-induced inflammatory response in microglial or immune cells via inhibition of NF-κB activity [29–32], as well as attenuate high glucose-stimulated activation of NF-κB [33]. The water-soluble Michael adduct of MCL, dimethylaminomicheliolide (DMAMCL, also known as ACT001), can slowly release MCL as a metabolite in plasma under physiological conditions [34]. DMAMCL can inhibit glioma cell growth in vitro and in vivo [35], and was recently approved for clinical trials in Australia to treat glioma tumor (trial ID: ACTRN12616000228482). DMAMCL also significantly prolongs the lifespan of a mouse model of human acute myelogenous leukemia (AML) through inhibiting NF-κB activity [36]. Moreover, DMAMCL is found to have very low side toxicities to animals which makes it a safe and suitable agent for a long-term treatment in vivo [35]. However, whether DMAMCL is suitable for anti-aging intervention in mammals, and whether it has an anti-aging effect via inhibition of NF-κB activity and can be a promising anti-aging agent remain totally unknown.

In the present study, we aimed to examine the effects of long-term administration of DMAMCL for 15 months with three different doses on the aging process in middle-aged male C57BL/6 mice, as well as long-term safety and toxicity. We presented evidences that chronic DMAMCL supplementation ameliorated or had little effect on some age-related degeneration and functional decline in mice without overt side effects. At a molecular level, we found DMAMCL treatment reduced serum levels of several important inflammatory cytokines, including IL-6, IL-1α, IL-1β, TNF-α, IFN-γ, and CXCL2, and suppressed NF-κB activity in several aged tissues. Our ﬁndings from this long-term administration study provide basic evidence to further study whether DMAMCL can be an effective anti-aging compound that prevents age-associated physiological decline.

## RESULTS

### Effects of DMAMCL treatment on body weight and survival rate

**Figure 1:**
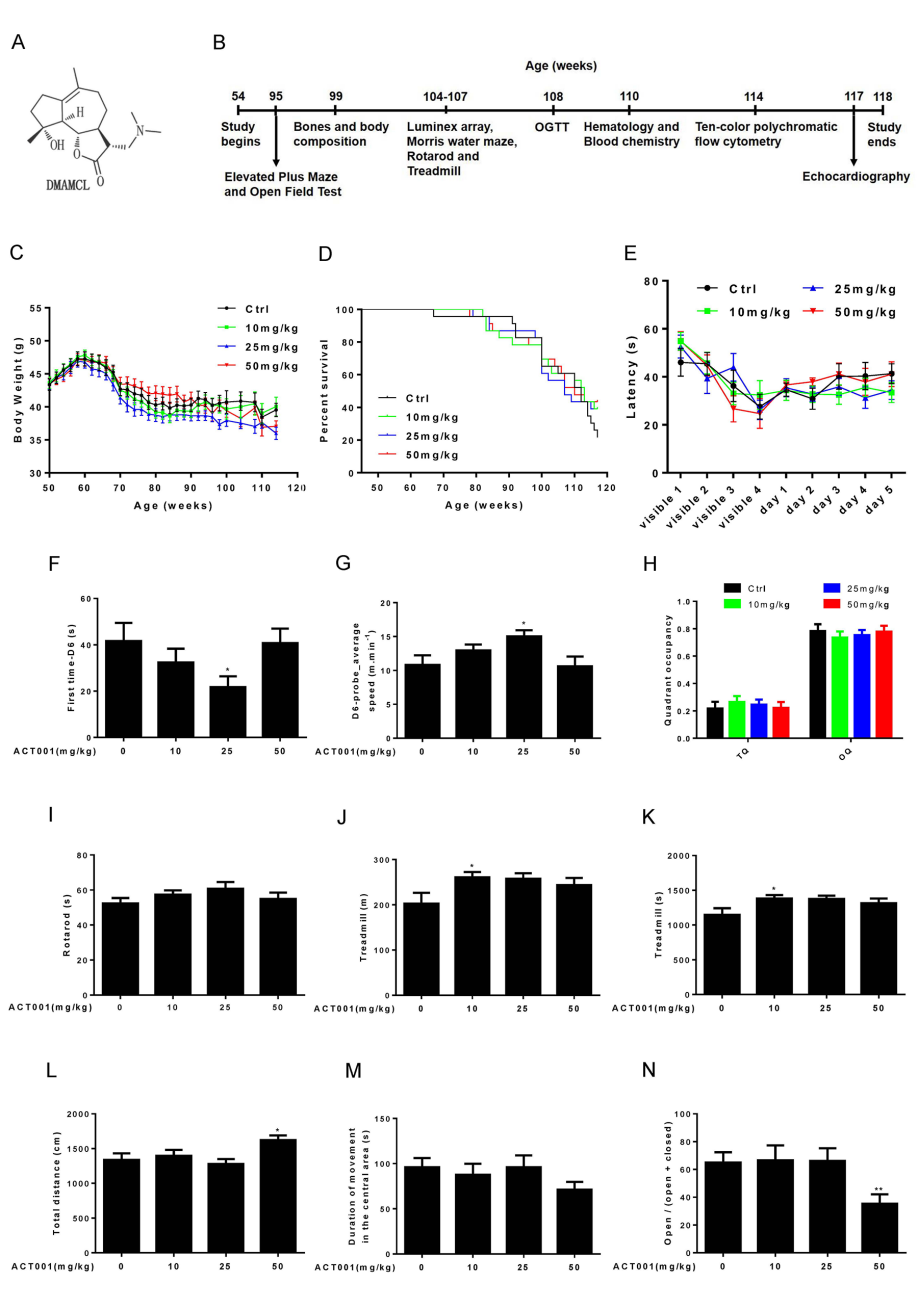
**Effects of DMAMCL treatment on body weight, survival rate, neurobehavioral phenotypes and physical performance.** (**A**) The chemical structure of DMAMCL. (**B**) A scheme showing the long-term DMAMCL administration and various analyses. DMAMCL treatment was initiated at 54 weeks, and the experiment lasted for 15 months. (**C**) Body weight. (**D**) Kaplan-Meier survival curves (n = 23 mice per experimental group x 4 groups: control, 10, 25, and 50 mg/kg/EOD). (**E-H**) Learning and memory ability was examined in the animals using the Morris water maze (n=12). (**E**) Latencies to find the platform. (**F**) The first time to find the platform during the probe trial at day 6. (**G**) Swimming speed at day 6. (**H**) Quadrant occupancy during the probe trial. TQ, target quadrant; OQ, other quadrants. (**I**) Time to fall from an accelerating rotarod (n=8-12). (**J** and **K**) Total distance (**J**) and time (**K**) ran on treadmill performance (n=9-10). (**L** and **M**) Total distance (**L**) and Duration of movement in the central area (**M**) in Open-field test (n=13-14 per group). (**N**) Open/ (open + closed) ratio in Elevated plus maze test (n=14 per group). Data are represented as the mean ± SEM. *P < 0.05 and **P < 0.01 compared with the control group (t-test two tailed).

To determine the effects of long-term DMAMCL administration on age-associated pathophysiology, we fed 1-year-old male C57BL/6 mice with standard control diet (SD) supplemented with DMAMCL by oral gavage every-other-day (EOD) for total 15 months (Fig. 1B). The chemical structure of DMAMCL was depicted in Fig. 1A. We tested three doses of DMAMCL, 10 (low), 25 (median), and 50 (high) mg/kg/EOD, from 12 months of age to 27 months of age (n = 23 mice per experimental group x 4 groups: vehicle control, 10, 25, and 50 mg/kg).

During 15-months intervention period, we carefully monitored the body weight of each group mice every other week. We found the average body weight of 10 mg/kg/EOD-fed mice was very similar to control mice during entire period (Fig. 1C, and Fig. S1A). However, the average body weight of 25 mg/kg/EOD-fed mice started to become less than control mice after 10 weeks of DMAMCL treatment and remained even more lesser thereafter (Fig. 1C, and Fig. S1B). Nevertheless, the 50 mg/kg/EOD-fed mice did not show dose-dependent loss of average body weight compared to the other three group’s mice. The average body weight of 50 mg/kg/EOD-fed mice was similar to control mice before 100 weeks and then started to become less than control mice thereafter (Fig. 1C, and Fig. S1AFig. S1C).

During 15 months treatment period, we also monitored the survival curves of control and DMAMCL-treated mice. There were no significant differences in the survival curves and median lifespan between control and three doses of DMAMCL-fed mice during entire treatment period (Fig. 1D, and Fig. S2A-C). However, we noted that the survival curves of control and DMAMCL-treated mice started to separate at late phase treatment. In fact, there was 8 to 10 mice survived in each of three doses of DMAMCL-treated mice, whereas only 5 mice left in control group at the end of 15 months treatment period before all mice were subjected to sacrifice (Fig. 1D, and Fig. S2A-C).

### Low or median doses of DMAMCL treatment improves some age**-**associated neurobehavioral phenotypes and physical performance

We determined a variety of physiological, biochemical, and molecular parameters in control and DMAMCL-fed mice. Aging is associated with the decline of cognitive functions, including impairments in learning and memory ability [37]. We examined learning and memory ability in mice using the Morris water maze. Mice were trained to find a platform hidden underneath the water surface in a constant location of the pool. We observed a slight improvement of the latency during 5 days training in low and median doses of DMAMCL-treated old mice compared to age-matched control mice but without reaching statistical signiﬁcance (Fig. 1E, and Fig. S3A, B). However, the latency in high dose DMAMCL-treated mice was very similar to control mice (Fig. 1E, and Fig. S3C). To test how accurately animals had learned the platform location, we removed the platform from the pool and analyzed the swim pattern of the animals at day 6. The first time to find platform location at day 6 (first time-D6) in median dose DMAMCL-treated old mice was significantly decreased relative to control mice (Fig. 1F). Consistent with this result, the average speed to find the probe at day 6 (D6-probe_average speed) in median dose DMAMCL-fed old mice was significantly increased compared to control mice (Fig. 1G). These results indicated that spatial learning and memory ability was significantly improved by chronic median dose DMAMCL treatment. We also observed a small but statistically insigniﬁcant improvement of spatial learning and memory ability in low dose DMAMCL-treated mice, and no improvement was found in high dose DMAMCL-treated mice, when compared with control mice (Fig. 1F, G). For the probe trial measures, there was a possible slight increase of target quadrant occupancy (TQ) and small decrease of other quadrant occupancy (OQ) in low and median doses DMAMCL-fed mice relative to control mice, however, it did not reach the significant difference (Fig. 1H). Again, there was no difference in TQ and OQ between high dose DMAMCL-treated mice and control mice (Fig. 1H).

We also tested motor coordination and balance using the accelerating rotarod. There was a possible improvement of latency to fall in low and median dose DMAMCL-treated mice compared to control mice but without reaching statistical signiﬁcant difference, and no difference between high dose DMAMCL-treated mice and control mice (Fig. 1I).

Exercise ability was also examined using treadmill test, in which mice were trained to run on the treadmill for 1 day, and then tested on the accelerating treadmill at second day. We observed an improvement in both running distance and time on treadmill in all doses DMAMCL-fed mice compared to control mice, but only low dose DMAMCL administration significantly enhanced exercise ability in old mice (Fig. 1J, K).

We further determined exploratory activity in an open field test, in which mice were allowed to explore freely a novel environment. High dose DMAMCL-treated mice showed significant increase in total distance traveled measures when compared with vehicle-treated mice (Fig. 1L), and there was no difference between low, median doses DMAMCL-treated mice and control mice. Additionally, the duration of movement in the central area was obviously reduced in high dose DMAMCL-fed mice relative to control mice though without reaching statistical significant difference (Fig. 1M). Furthermore, the ratio of open/ (open + closed) measured by elevated plus maze in high dose DMAMCL-treated mice was also significantly decreased when compared with control mice (Fig. 1N). These results suggest that long-term administration of high dose DMAMCL may cause side effect to induce anxious behavior in mice. However, there was no difference in duration of movement in the central area and the ratio of open/ (open + closed) between low, median doses DMAMCL-fed mice and control mice, which indicated that long-term low and median doses DMAMCL treatment did not induce anxiety in mice.

### The effects of DMAMCL treatment on bones and body composition

**Figure 2:**
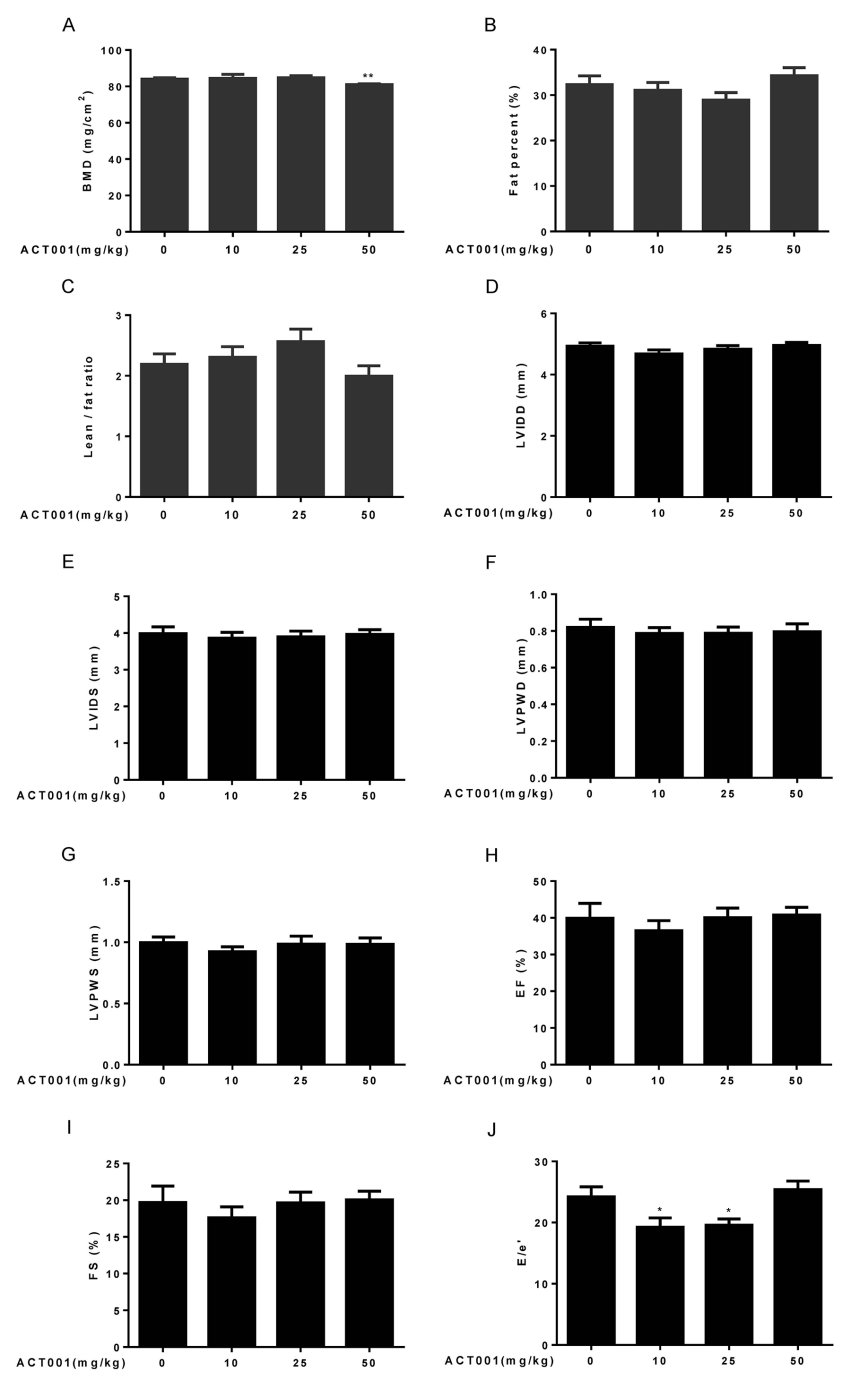
**Assessment of bone structure, body composition, and cardiological analyses.** (**A-C**) Bone mineral density (BMD) and body composition were examined by dual-energy X-ray absorptiometry (DEXA) (n=10). (**A**) Bone mineral density. (**B**) Fat percent. (**C**) Lean/fat ratio. (**D-J**) Heart dimensions and functions were assessed using an echocardiography (n=6-8). Dimensions of (**D**) left ventricular internal diameter in diastole, (**E**) left ventricular internal diameter in systole, (**F**) left ventricular posterior wall in diastole, and (**G**) left ventricular posterior wall in systole were determined. (**H**) Ejection fraction (%). (**I**) Fractional shortening. (**J**) E/e’ ratio. Data are represented as the mean ± SEM. *P < 0.05 and **P < 0.01 compared with the control group (t-test two tailed).

Age-associated progressive bone loss results in osteoporosis, reduced bone strength, and fractures [38]. We measured bone mineral density (BMD) by dual-energy X-ray absorptiometry (DEXA). Low and median doses of DMAMCL supplementation had no any measurable effect on BMD compared to control mice. However, high dose DMAMCL treatment led to a small but signiﬁcant decrease in BMD relative to control mice (Fig. 2A), which suggested that long-term supplementation of high dose DMAMCL might cause bone loss in mice.

Aging is associated with the increase in proportional body fat mass and decrease in relative lean mass (fat and lean mass adjusted to body weight) [39]. Therefore, we also detected lean and fat masses by DEXA. The results showed that fat percentage reduced and lean/fat ratio elevated in low and median doses DMAMCL-treated mice compared with control mice but without reaching statistical difference (Fig. 2B, C). Conversely, fat percentage increased and lean/fat ratio decreased in high dose DMAMCL-treated mice compared to control mice, also without reaching statistical difference (Fig. 2B, C).

### DMAMCL treatment has no apparent effect on age**-**associated changes in cardiac structure but improves its diastolic function

Aging is associated with signiﬁcant changes in heart structure and function [40]. To examine cardiac aging phenotypes, we performed echocardiography to measure heart dimensions and functions. Age-related increases in some heart dimensional echocardiography measures, including left ventricular internal diameter in diastole (LVIDD), left ventricular internal diameter in systole (LVIDS), left ventricular posterior wall in diastole (LVPWD), and left ventricular posterior wall in systole (LVPWS) might be slightly decreased by low dose DMAMCL treatment without reaching statistical difference, while median and high doses DMAMCL treatment had no any measurable effects on these parameters when compared with vehicle treatment (Fig. 2D-G).

Aging is associated with decrease in some heart functional echocardiography measures, including ejection fraction (EF) and fractional shortening (FS) [41]. Our results showed that DMAMCL administration did not measurably improve these cardiac functional declines, instead low dose DMAMCL treatment marginally reduced EF and FS but without reaching statistical significant difference (Fig. 2H, I). E/e’ is a very important index to evaluate the function of diastolic myocardium and estimate the left ventricular filling pressure (LVFP), and E/e’ ratio increases with age [42]. We observed a significant reduction of E/e’ ratio in both low and median doses DMAMCL-fed mice relative to control mice, while the ratio remained unchanged in high dose DMAMCL-treated mice (Fig. 2J). These results suggest that long-term administration of low and median doses DMAMCL may improve cardiac diastolic function in aged mice.

### DMAMCL treatment has little effect on some basic hematological parameters

**Figure 3:**
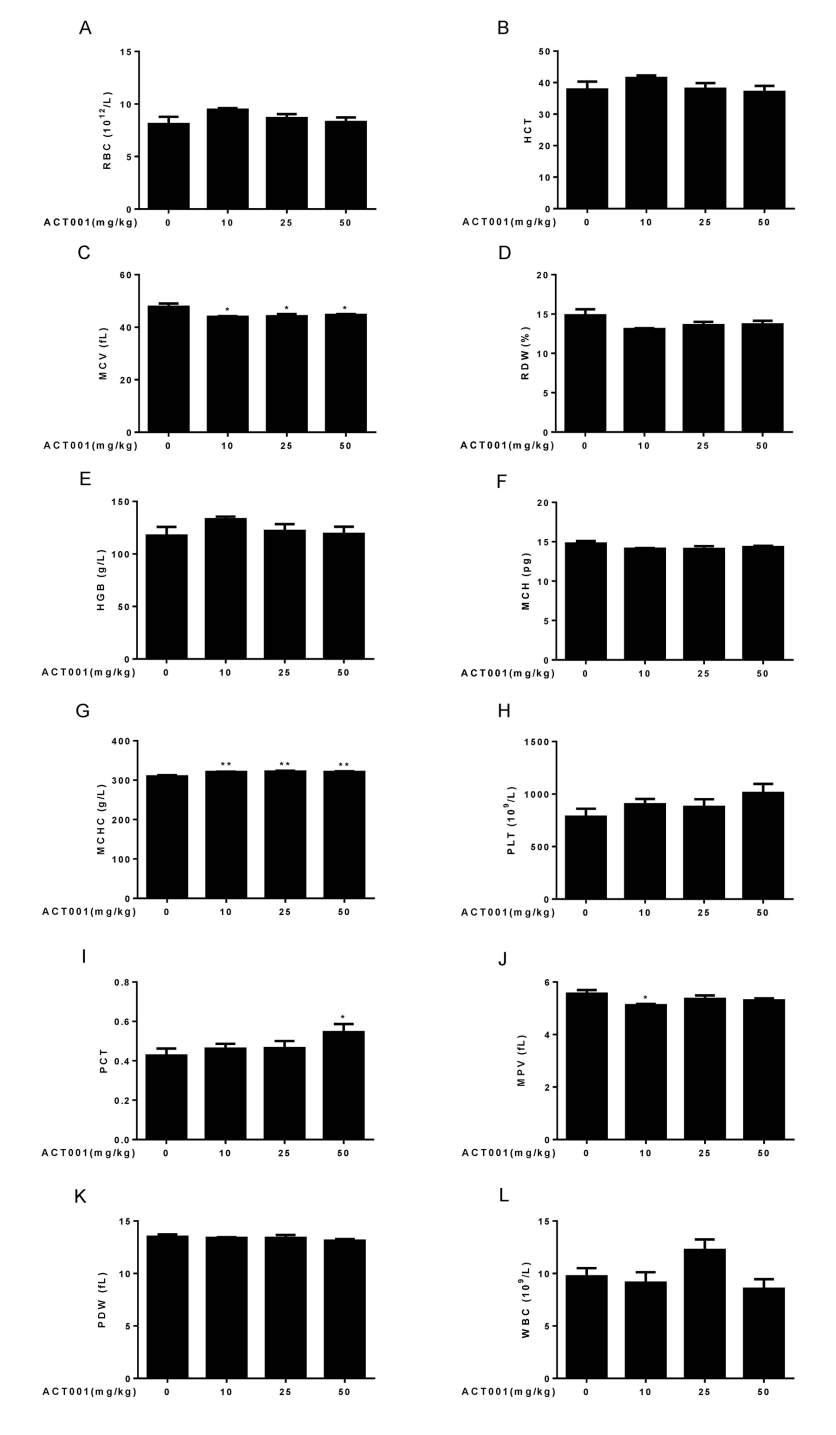
**Hematological analyses.** Number and size of the different blood cell types were determined using a hematology analyzer (n=10). (**A**) RBC count. (**B**) HCT. (**C**) MCV. (**D**) RDW. (**E**) HGB. (**F**) MCH. (**G**) MCHC. (**H**) PLT. (**I**) PCT. (**J**) MPV. (**K**) PDW. (**L**) WBC. Data are represented as the mean ± SEM. *P < 0.05 and **P < 0.01 compared with the control group (n=10, t-test two tailed).

Aging is associated with an altered cellular composition of the peripheral blood in C57BL/6 mice [41,43,44]. Thus, we detected basic hematological assessments in these old mice. It is known that red blood cell counts (RBC), hematocrit (HCT), hemoglobin concentrations (HGB), and mean corpuscular hemoglobin concentrations (MCHC) significantly decrease, whereas RBC distribution width (RDW) significantly increases in old mice. In addition, mean corpuscular volume (MCV) slightly increases with aging, and mean corpuscular hemoglobin content (MCH) remains almost unchanged during aging [41,43,44]. Chronic low dose DMAMCL treatment caused an overall increase of RBC, HCT, and HGB, and all doses DMAMCL treatment significantly elevated MCHC, when compared with vehicle-treated old mice (Fig. 3A, B, E, G). Besides, all doses DMAMCL treatment significantly mitigated MCV, and caused an overall decrease of RDW, when compared with control old mice (Fig. 3C, D). MCH remained almost unchanged by DMAMCL treatment (Fig. 3F).

It is known that platelet counts (PLT), plateletcrit (PCT), mean platelet volume (MPV), and platelet distribution width (PDW) significantly increase in aged mice [41,43,44]. Our results revealed that all doses DMAMCL treatment promoted PLT but without reaching statistically significant difference (Fig. 3H). In addition, low and median doses DMAMCL treatment slightly increased PCT, but high dose DMAMCL significantly elevated PCT, when compared with control old mice (Fig. 3I). In contrast, all doses DMAMCL treatment resulted in a possible slight reduction of MPV and PDW, but only low dose DMAMCL treatment significantly decreased MPV, when compared with control aged mice (Fig. 3J, K).

White blood cell counts (WBC) is possibly increased in aged mice but without reaching statistically significant difference [41,43,44]. The elevation of WBC was observed in median dose DMAMCL-fed mice, whereas low and high doses DMAMCL treatment reduced WBC when compared with control old mice (Fig. 3L), but all results did not reach statistically significant difference.

### DMAMCL treatment has little effect on a subset of age**-**associated immune changes

**Figure 4:**
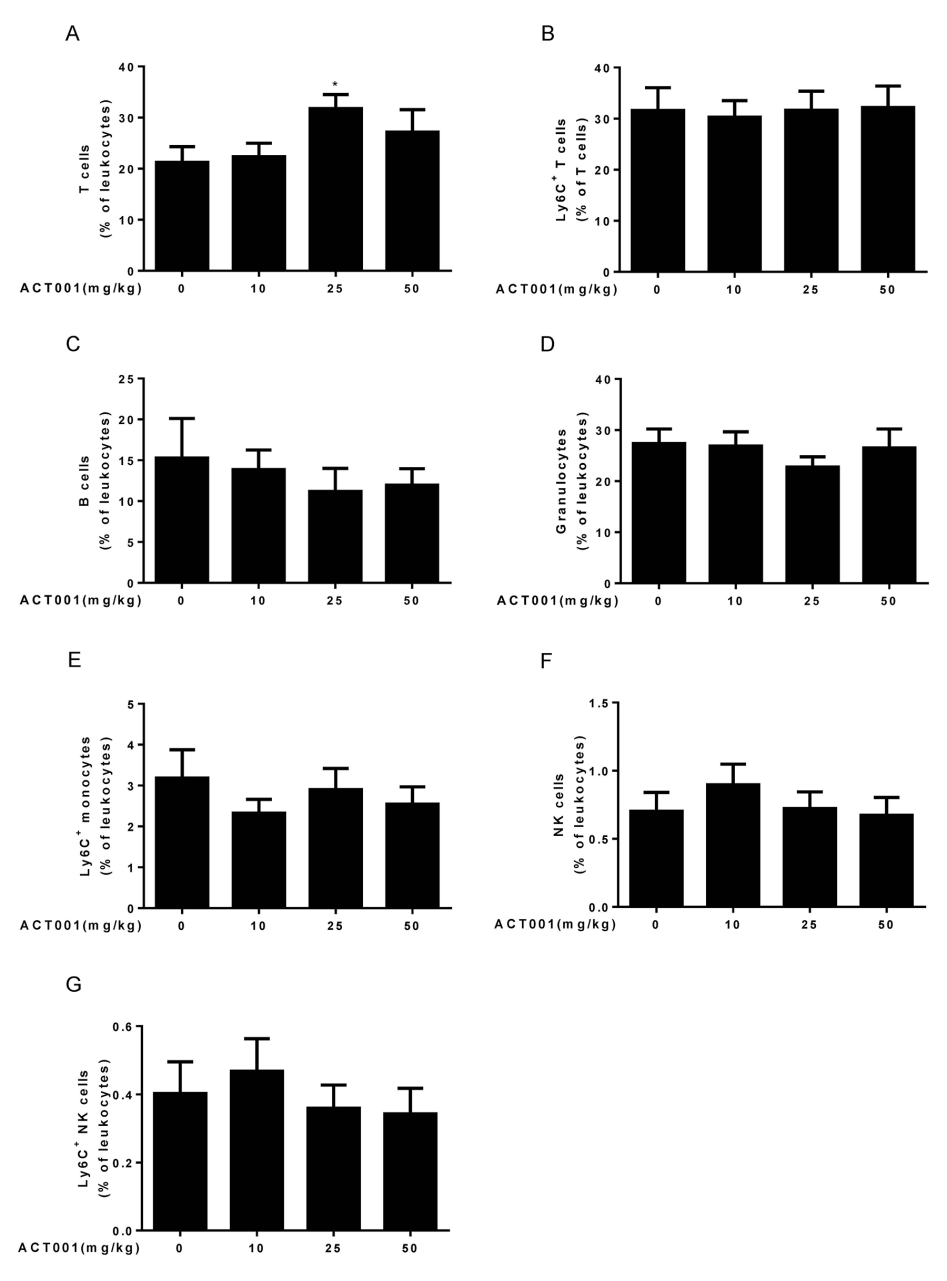
**Aging-associated immunological analyses.** Peripheral blood leukocytes were analyzed using a ten-color polychromatic flow cytometer. (**A**) T cells. (**B**) Ly6C^+^ T cells. (**C**) B cells. (**D**) Granulocytes. (**E**) Ly6C^+^ monocytes. (**F**) Natural killer (NK) cells. (**G**) Ly6C^+^ NK cells. Data are represented as the mean ± SEM. *P < 0.05 compared with the control group (n=7-10, t-test two tailed).

Aging is associated with immune system dysfunction. Immune aging phenotypes affect the lymphocyte which include decreased numbers of T, B, and natural killer (NK) cells, and increased frequencies of monocytes and granulocytes [45]. To examine whether DMAMCL administration affect these immune aging phenotypes, we performed ten-color polychromatic ﬂow cytometry and quantiﬁed a subset of immune cell populations in DMAMCL-treated and control old mice. Our results showed that all doses DMAMCL treatment caused an overall increase of T cell frequencies, among these, median dose DMAMCL significantly increased T cell proportions relative to control mice (Fig. 4A). Aging is associated with increase in the proportion of T cells expressing memory markers, such as CD44 and/or Ly6C [41,44]. Ly6C^+^ T cells remained almost unaffected by all doses DMAMCL treatment, though a possible marginal decrease in low dose DMAMCL-treated mice (Fig. 4B).

Age-related decrease of B cells was not reversed by all doses DMAMCL supplementation. Conversely, all doses DMAMCL treatment further reduced B cell frequencies compared to vehicle treatment but without reaching statistically significant difference (Fig. 4C). Granulocytes frequencies remained almost unaffected by low and high doses DMAMCL treatment, but alleviated by median dose DMAMCL treatment though without reaching statistically significant difference (Fig. 4D). Age-related increase of Ly6C^+^ monocytes frequencies was attenuated by all doses DMAMCL administration, though without reaching statistically significant difference (Fig. 4E). In addition, age-related decrease of NK cells and Ly6C^+^ NK cells frequencies were recovered by low dose DMAMCL treatment, though without reaching statistically significant difference (Fig. 4F, G). Altogether, these results revealed that DMAMCL treatment opposed a subset of immune aging phenotypes within the lymphocytes (i.e., effects on T cells, granulocytes, Ly6C^+^ monocytes, NK cells, and Ly6C^+^ NK cells).

### DMAMCL treatment significantly mitigates many myocardial zymogram and has little effect on other age**-**associated changes in clinical chemistry parameters

**Figure 5:**
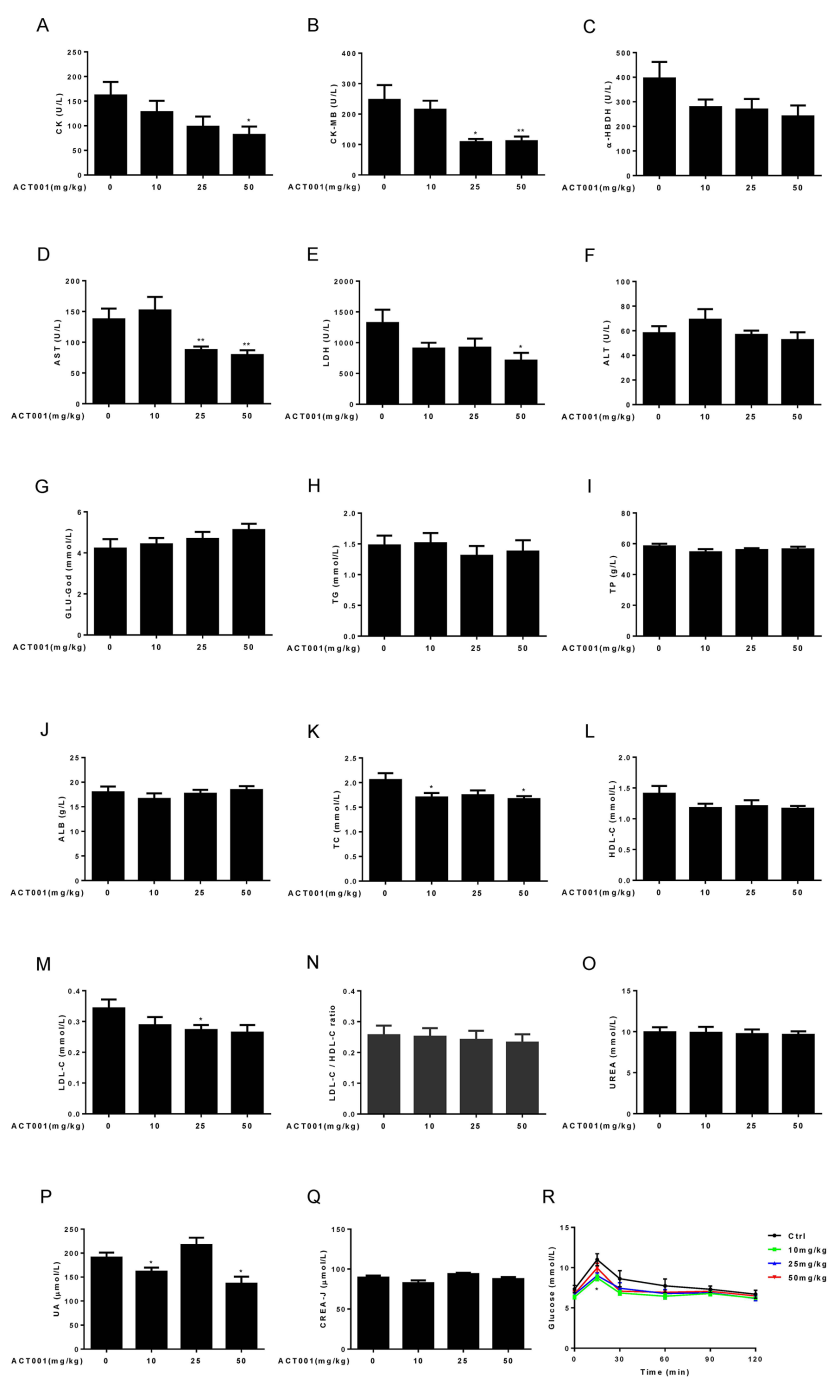
**Clinical chemistry parameters and assessment of glucose homeostasis.** (**A-E**) Myocardial zymogram parameters, including (**A**) Creatine kinase (CK), (**B**) Creatine kinase-MB form (CK-MB), (**C**) α-hydroxybutyric dehydrogenase (α-HBDH), (**D**) AST, and (**E**) LDH were determined in mice. (**F-Q**) Plasma concentrations of (**F**) ALT, (**G**) GLU-God, (**H**) triglycerides (TG), (**I**) total protein (TP), (**J**) ALB, (**K**) total cholesterol (TC), (**L**) HDL-C, (**M**) LDL-C, (**O**) UREA, (**P**) UA, and (**Q**) CREA-J were measured in animals. (**N**) LDL-C/HDL-C ratio was calculated (n=7-10). (**R**) Blood glucose concentration during an OGTT (n =10). Data are represented as the mean ± SEM. *P < 0.05 and **P < 0.01 compared with the control group (t-test two tailed).

Aging is associated with many changes in clinical chemistry parameters [41,44], which include the increases of aspartate aminotransferase (AST, or GOT), lactate dehydrogenase (LDH), alanine aminotransferase (ALT, or GPT), triglycerides (TG), total protein (TP), and urea (UREA), as well as the decreases of glucose (GLU-God) and albumin (ALB). To determine whether DMAMCL supplementation has any effect on these age-associated changes, we performed clinical chemistry studies (plasma taken from mice in the fasting state) in DMAMCL-treated and control old mice. We observed that DMAMCL treatment had apparent effect on most myocardial zymogram parameters (Fig. 5A-E). Almost all doses DMAMCL treatment partially or significantly alleviated creatine kinase (CK), creatine kinase-MB form (CK-MB), α-hydroxybutyric dehydrogenase (α-HBDH), AST, and LDH. Moreover, the inhibition effects of DMAMCL on these parameters might display dose-dependent manner at some extent (Fig. 5A-E). We noted a possible slight decrease of ALT in median and high doses DMAMCL-fed mice compared to control mice (Fig. 5F), but low dose DMAMCL treatment increased ALT relative to control mice without reaching statistically significant difference.

DMAMCL administration might dose-dependently reverse the age-related decrease of GLU-God but without reaching statistically significant difference (Fig. 5G). Almost all doses DMAMCL treatment might slightly reduce the age-related increases of TG (except low dose DMAMCL) and TP but without reaching statistically significant difference (Fig. 5H, I). ALB remained almost unchanged in median and high doses DMAMCL-treated mice relative to control mice and might be slightly decreased by low dose DMAMCL treatment (Fig. 5J).

All doses DMAMCL treatment partly or significantly mitigated total cholesterol (TC), high-density lipoprotein cholesterol (HDL-C), and low-density lipoprotein cholesterol (LDL-C) compared to control old mice (Fig. 5K-M). A possible slight decrease of LDL-C/HDL-C ratio was observed in DMAMCL-treated mice relative to control mice but without reaching statistically significant difference (Fig. 5N).

Age-related increase of UREA might be very slightly attenuated by DMAMCL treatment but without reaching statistically significant difference (Fig. 5O). Low and high doses DMAMCL administration significantly reduced UA level compared to control mice, instead, median dose DMAMCL increased UA level but without reaching statistically significant difference (Fig. 5P). Similarly, creatinine (CREA-J) level was slightly alleviated by low and high doses DMAMCL supplementation compared to control mice but without reaching statistically significant difference, and remained unchanged in median dose DMAMCL-fed mice (Fig. 5Q).

### DMAMCL treatment significantly improves glucose homeostasis in aged mice

Aging is strongly associated with insulin resistance and type 2 diabetes [46]. To investigate whether DMAMCL treatment has any effect on age-related changes in glucose tolerance, we performed an oral glucose tolerance test (OGTT) in control and DMAMCL-fed old mice (after 6 h of fasting). The basal fasting blood glucose levels in all doses DMAMCL-treated mice were lower than in control mice (Fig. 5R, and Fig. S4A, B). More importantly, we observed that low and median doses DMAMCL-fed mice exhibited a signiﬁcant decrease in blood glucose levels at the OGTT peak (15 min) and subsequent inter-individual variance of glucose levels compared to control mice (Fig. 5R, and Fig. S4A), which indicated a higher rate of glucose clearance in DMAMCL-treated mice. Although high dose DMAMCL treatment also mitigated the glucose levels at OGTT, the difference did not reach statistical significance (Fig. 5R, and FFig. S4B).

### DMAMCL treatment significantly alleviates several important age**-**related inﬂammatory cytokines levels in old mice

**Figure 6:**
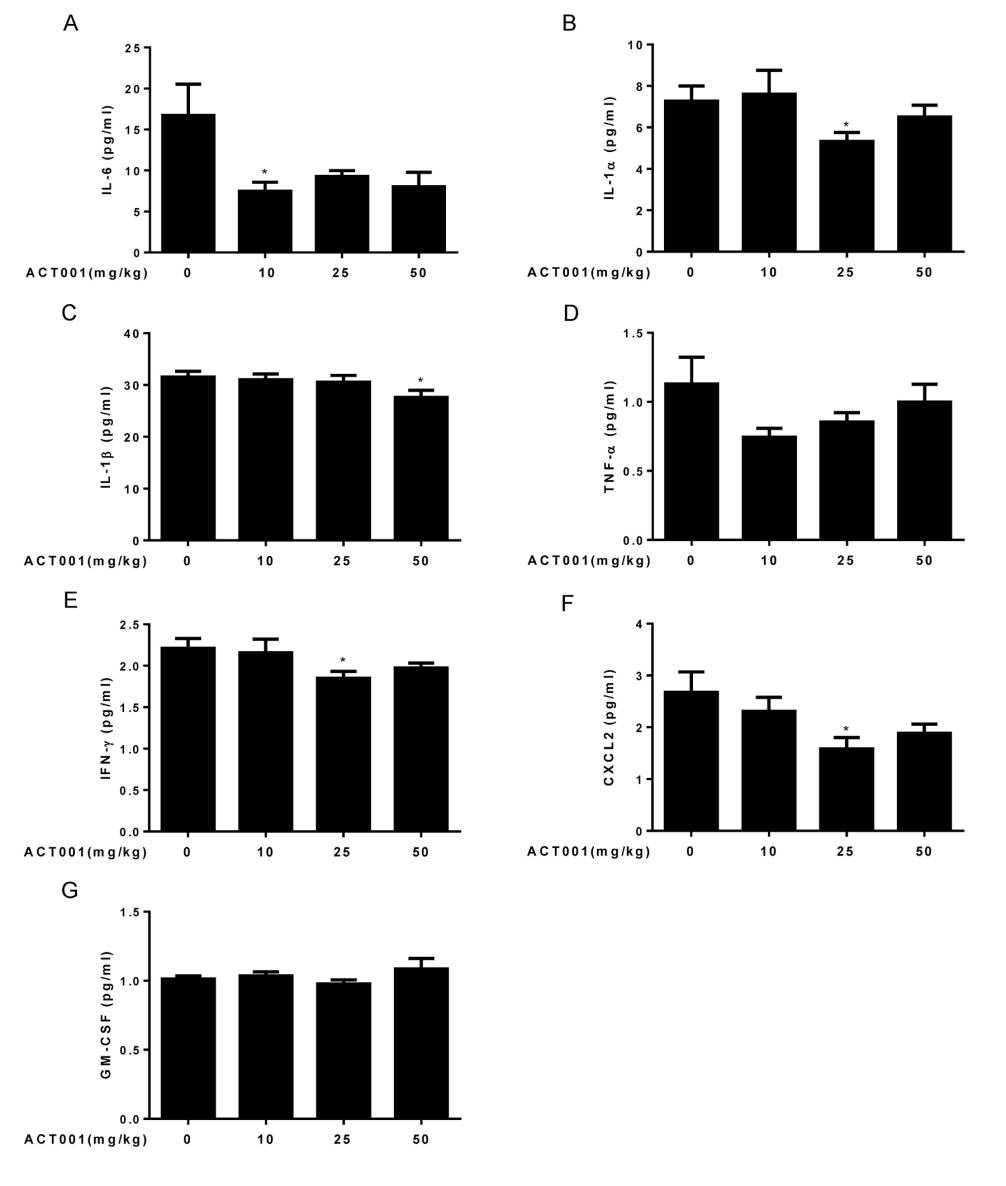
**DMAMCL treatment represses age-related blood levels of inﬂammatory cytokines.** The blood levels of seven inﬂammatory cytokines, including (**A**) IL-6, (**B**) IL-1α, (**C**) IL-1β, (**D**) TNF-α, (**E**) IFN-γ, (**F**) CXCL2, and (**G**) GM-CSF were measured using a mouse magnetic Luminex screening assay (n=10). Data are represented as the mean ± SEM. *P < 0.05 compared with the control group (n=10, t-test two tailed).

Aging is associated with the increase of serum levels of some inﬂammatory cytokines such as IL-6, TNF-α, interferon (IFN)-γ, IL-18, IL-8, and IL-1β in the older subjects compared to the young subjects, and strongly correlate with increased risk of morbidity and mortality in the older subjects [4,5]. NF-κB is a master transcription factor to regulate the expression of these inﬂammatory cytokines [6]. Since DMAMCL can inhibit NF-κB activity, we sought to investigate whether long-term administration of DMAMCL could reduce the serum levels of inﬂammatory cytokines in old mice. Therefore, we performed Luminex array to measure the blood levels of seven inﬂammatory cytokines, including IL-6, IL-1α, IL-1β, TNF-α, IFN-γ, CXCL2, and GM-CSF in control and DMAMCL-treated old mice. All doses DMAMCL treatment significantly decreased IL-6 levels compared to control mice (Fig. 6A). Median and high doses DMAMCL treatment significantly or partly inhibited IL-1α levels, while there was no significant difference between low dose-treated and vehicle-treated mice (Fig. 6B). Median and high doses DMAMCL administration slightly or significantly inhibited IL-1β levels, respectively, whereas low dose DMAMCL treatment had no apparent effect on IL-1β level compared to control mice (Fig. 6C). All doses DMAMCL supplementation restrained TNF-α levels compared to control mice, but only low dose DMAMCL treatment reached one-tail t test statistical significance (Fig. 6D). Median and high doses DMAMCL treatment also significantly impeded IFN-γ levels (one tail or two tail t test) compared to control mice (Fig. 6E). In addition, all doses DMAMCL administration hampered CXCL2 levels compared to control mice, in which median and high doses DMAMCL treatment reached two-tail or one-tail t test statistical significance, respectively (Fig. 6F). All doses DMAMCL treatment had no apparent effect on GM-CSF levels relative to control mice (Fig. 6G). Collectively, these results suggested that long-term DMAMCL supplementation could reduce several important age-related inﬂammatory cytokines levels in old mice.

### DMAMCL treatment inhibits NF**-**κB activities in several tissues and organs in old mice

**Figure 7:**
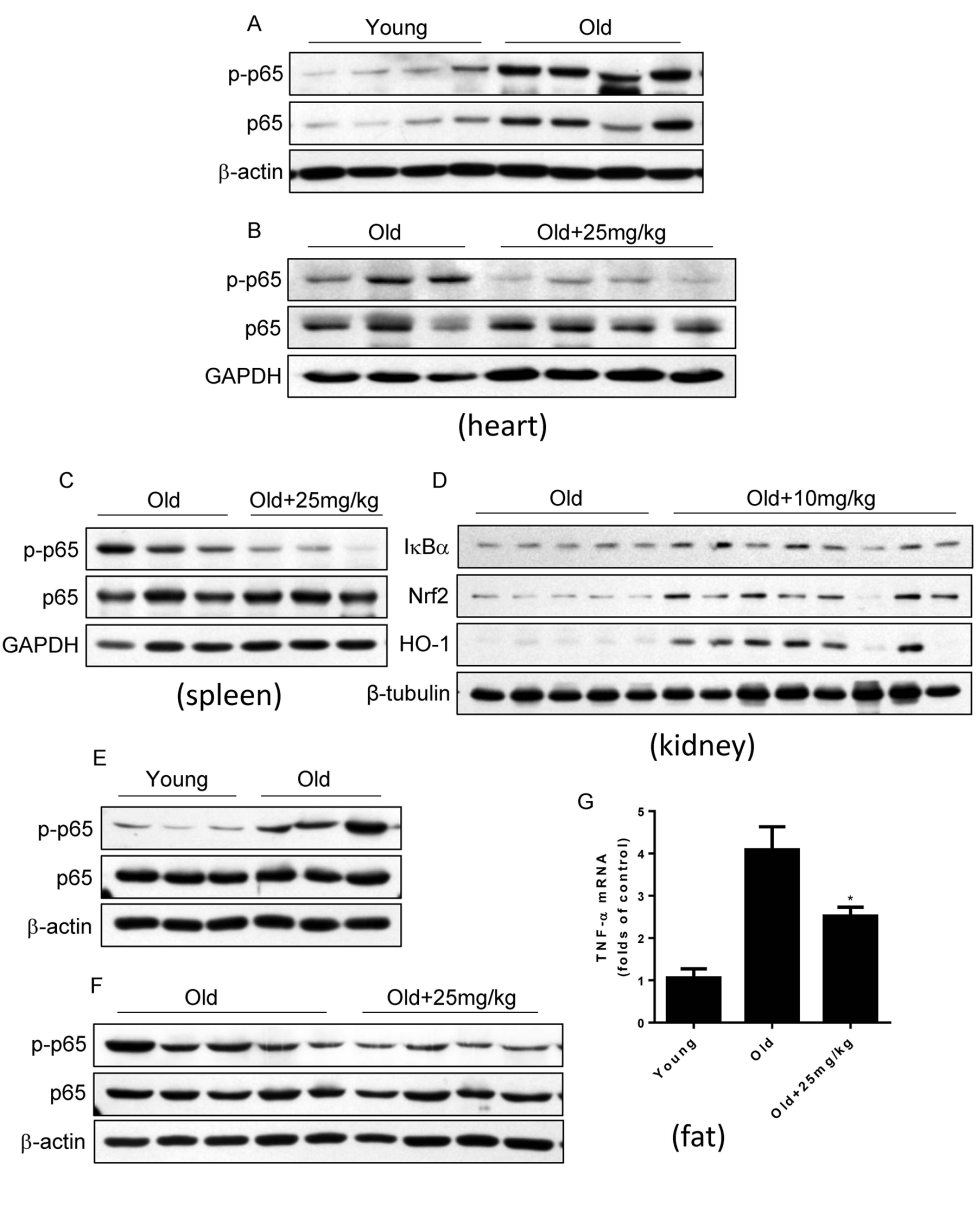
**DMAMCL treatment suppresses NF-κB activities in multiple tissues and organs in old mice.** The total protein was isolated from the heart, spleen, kidney, and fat tissues. The p-p65 and p65 protein expression levels in heart (**A** and **B**), and in spleen (**C**), the expression levels of IκBα, HO-1 and Nrf2 in kidney (**D**), and p-p65 and p65 protein expression levels in fat (**E** and **F**), were examined by Western blot (n=3-8). (**G**) TNF-α mRNA levels in fat tissue were detected by qRT-PCR. Data are represented as the mean ± SEM. *P < 0.05 compared with the control group (t-test two tailed).

Last, we detected whether DMAMCL treatment could inhibit NF-κB activities in some tissues and organs in aged mice by western blot. Agreed with previous reports [7–9], we confirmed that NF-κB activity in heart increased in old mice relative to the young mice which characterized by the large rise of p-p65 levels (Fig. 7A). Importantly, median and high doses DMAMCL administration sufficiently blunted NF-κB activities in heart which indicated by the large decrease of p-p65 levels when compared with control old mice (Fig. 7B, and Fig. S5A). MCL is reported to promote antioxidant protein heme oxygenase-1 (HO-1) expression by enhancing NF-E2-related factor 2 (Nrf2) activity [32]. We also observed that DMAMCL treatment enhanced HO-1 expression in heart (Fig. S5A). Likewise, all doses DMAMCL treatment significantly suppressed NF-κB activities in spleen compared to control old mice (Fig. 7C, and Fig. S5B, C). Furthermore, low and high doses DMAMCL treatment also hindered NF-κB activities and augmented Nrf2 and HO-1 expression levels in kidney when compared with control old mice (Fig. 7D, and Fig. S5D).

Adipose tissue inflammation which can be described by the NF-κB activation and inflammatory cytokines such as TNF-α upregulation is strongly associated with glucose intolerance, insulin resistance and type 2 diabetes [47,48]. The finding that DMAMCL supplementation improved glucose homeostasis in old mice at OGTT test prompted us to explore whether DMAMCL treatment can attenuate adipose tissue inflammation. We observed that NF-κB activity in adipose tissue significantly increased in old mice compared to the young mice (Fig. 7E). Importantly, median dose DMAMCL treatment effectively suppressed NF-κB activity in adipose tissue relative to control old mice (Fig. 7F). Correlating with NF-κB activation in old adipose tissue, TNF-α mRNA level also largely augmented in old adipose tissue when compared with the young one (Fig. 7G). Similarly, median dose DMAMCL treatment significantly mitigated TNF-α mRNA level compared to control old mice (Fig. 7G). Taken together, these results suggest that DMAMCL treatment inhibits NF-κB activities in multiple tissues and organs in old mice.

## DISCUSSION

NF-κB activity increases with aging in a variety of tissues of mammals [7–13] and contributes to a number of age-related disorders and pathologies [14–21]. Mounting evidences demonstrate a causal relationship between NF-κB activation and aging process, and NF-κB inhibition either by genetic impairment or by pharmacological agents has been shown to mitigate many age-related symptoms and extend healthspan and lifespan in diverse animal models [49]. However, the small molecular compounds that can target to NF-κB activity and is suitable for aging intervention in mammals is currently lacking. DMAMCL is reported to be able to inhibit NF-κB activities at diverse biological or pathophysiological conditions [29–33,36]. In addition, DMAMCL is water soluble, is easy to cross blood brain barrier (BBB), and is proved to have very low side toxicities to animals even for a long-term treatment in vivo [29–36]. These properties make DMAMCL to be a suitable agent for aging intervention. In this study, we used three different doses of DMAMCL to test whether DMAMCL treatment slows mammalian aging rates by examining its effects on a wide range of functional and structural aging phenotypes in middle-aged C57BL/6 male mice. We demonstrate that long-term administration of low or median dose of DMAMCL mitigate or has little effect on some age-associated physiological decline in mice, while high dose DMAMCL may cause a limited side effects.

We found that 15-months-long supplementation of median dose of DMAMCL reduced average body weight of mice (Fig. 1C, and Fig. S1A-C). Consistent with this result, the fat percent was decreased and lean/fat ratio was increased in median dose DMAMCL-treated mice compared to control mice (Fig. 2B, C), and TG was also reduced in median and high doses DMAMCL-treated mice (Fig. 5H). These results suggest that long-term administration of DMAMCL may affect fat metabolism in mice. High dose DMAMCL treatment caused small but signiﬁcant decrease in BMD, but this side effect was not seen in low and median doses DMAMCL-fed mice (Fig. 2A).

We observed that low and median doses DMAMCL treatment insignificantly or significantly improved spatial learning and memory ability (Fig. 1E-H), motor coordination and balance (Fig. 1I), and physical performance (Fig. 1J, K), and had no effect on exploratory activity (Fig. 1L-N), when compared with control old mice. These results suggest that long-term administration of low or median dose DMAMCL may mitigate the decline of neurological functions and exercise ability. NF-κB activation and inflammation have been linked to neurodegenerative diseases, such as Alzheimer’s disease and Parkinson’s disease [18–20]. We previously reveal that MCL and its derivative DMAMCL are able to repress LPS-induced neuroinflammatory response in microglial cells and in mice via inhibition of NF-κB activity [32]. In addition, DMAMCL is water soluble and is easy to cross BBB [29–36]. Taken together, these results strongly suggest that it is deserve to further investigate whether DMAMCL has the effect on neurodegenerative diseases including Alzheimer’s disease and Parkinson’s disease. Nevertheless, we also noted that high dose DMAMCL may induce anxious behavior in mice (Fig. 1M, N), but this side effect can be avoided by using lower dose of DMAMCL.

DMAMCL treatment had no effect on age-associated changes in cardiac structure and cardiac function-related EF and FS (Fig. 2D-I), but low and median doses DMAMCL treatment significantly reduced E/e’ ratio (Fig. 2J). E/e’ ratio is increasingly recognized as an important marker to estimate left ventricular filling pressure (LVFP) [50], left ventricular diastolic function (LVDF) [51], acute and chronic diastolic heart failure (DHF) [52,53], acute myocardial infarction (AMI) and acute coronary syndrome (ACS) [51,54]. In line with this result, DMAMCL treatment also partly or significantly attenuated most myocardial zymogram parameters, including CK, CK-MB, α-HBDH, AST, and LDH (Fig. 5A-E), which are used clinically to diagnose AMI and myocardial injury. Collectively, these results strongly suggest that DMAMCL may have cardiac protection effect and it is worth to further exploit whether DMAMCL plays a role in various cardiac dysfunctions.

The reduced RBC, HCT, HGB and MCHC in aged mice were elevated (Fig. 3A, B, E, G), and the increased RDW in old mice were alleviated (Fig. 3D), by DMAMCL treatment at different extent, respectively. DMAMCL administration also increased PLT and PCT, but mitigated MPV and MCV (Fig. 3C, H-J). The implications of these changes in hematological parameters need to be further explored.

The aging-associated immune changes, such as the decreased numbers of T and NK cells, and increased frequencies of granulocytes and monocytes, were reversed by DMAMCL treatment at different extent (Fig. 4A, D-G). Given that NF-κB signaling plays critical role in modulating immunity and DMAMCL is an inhibitor of NF-κB activity, the long-term administration of DMAMCL seems to improve immune aging phenotypes a little bit in old mice, instead of suppressing immune system from our results.

Long-term DMAMCL treatment partially or significantly attenuated TC, HDL-C, and LDL-C (Fig. 5K-M). These results suggest that long-term supplementation of DMAMCL may affect cholesterol metabolism in mice. DMAMCL treatment had no significant effect on TP and ALB (Fig. 5I, J). Long-term DMAMCL treatment partly or significantly lowered AST, ALT (Fig. 5D, F), and UA as well as CREA-J (Fig. 5P, Q), instead of causing toxicities to liver and kidney. These results indicate that DMAMCL treatment appears to ameliorate the functional decline of liver and kidney a little bit in aged mice, as well as the safety of long-term administration of DMAMCL.

We observed that all doses DMAMCL treatment partly or significantly reduced blood glucose levels in old mice at OGTT test (Fig. 5R, and Fig. S4A, B). Moreover, DMAMCL administration also suppressed adipose tissue inflammation in old mice which is associated with age-related insulin resistance and type 2 diabetes (Fig. 7E-G). These results imply that DMAMCL treatment may improve insulin sensitivity, glucose homeostasis, and prevent type 2 diabetes during aging. Therefore, it is deserved to further investigate whether DMAMCL plays a preventive effect on age or obesity-related insulin resistance and type 2 diabetes.

The chronic, low-grade inﬂammation is one of the major hallmarks of aging, termed ‘inflammaging’. Inﬂammaging has been assessed by measuring a number of inﬂammatory cytokines, chemokines, and acute-phase proteins, in the blood of older subjects [4,5]. Among these inflammatory markers, age-related increase of IL-6, TNF-α, IL-1β, and IFN-γ, etc., are strong predictors of morbidity and mortality in older subjects [55–58]. We found that long-term DMAMCL supplementation reduced IL-6, IL-1α, IL-1β, TNF-α, IFN-γ, and CXCL2 blood levels at different extents by different doses of DMAMCL in old mice (Fig. 6A-F). At molecular level, we further proved that DMAMCL treatment inhibited NF-κB activities in several tissues and organs in old mice (Fig. 7A-G, and Fig. S5A-D). Taken together, these results indicate that long-term administration of DMAMCL suppresses NF-κB-mediated systemic inflammation in aged mice.

It should be mentioned that all doses of DMAMCL administration did not generate an obvious toxicities and serious side effects throughout the 15-months-long intervention period, except high dose DMAMCL treatment might induce anxiety (Fig. 1M, N) and decrease BMD (Fig. 2A) in old mice. All doses of DMAMCL supplementation also did not increase mortality rate. In fact, all doses of DMAMCL treatment including high dose DMAMCL might increase survival rate, since 8 to 10 mice survived in each of three doses of DMAMCL-treated groups, whereas only 5 mice left in control group at the end of 15 months intervention period before all mice were subjected to sacrifice (Fig. 1D, and Fig. S2A-C). These results suggest that DMAMCL administration is well-tolerated and the long-term DMAMCL treatment is safe in mice, which is agreement with previous report [35]. Besides inhibition of NF-κB activity, pyruvate kinase M2 (PKM2) is recently identified as another target of DMAMCL [59]. DMAMCL can selectively activate PKM2 through the covalent binding to the conserved cysteine424 (C424) residue of PKM2. This interaction induces the irreversible tetramerization of PKM2. Via fixation PKM2 in its active tetrameric form, DMAMCL signiﬁcantly suppresses the leukemia cells growth. Although DMAMCL can target PKM2, however, PKM2 is mostly upregulated in cancer cells and not in normal cells, this fact confers DMAMCL much more specificity to target tumor cells, not to normal cells. In addition, DMAMCL (also known as ACT001) is currently undergoing clinical trials in Australia to treat glioblastoma patients (trial ID: ACTRN12616000228482). The phase 1 dose-escalation study of ACT001 in patients with recurrent glioblastoma and other advanced solid tumors results showed satisfactory bioavailability and preliminary evidence of anti-tumor activity in a subset of patients at well-tolerated doses [60]. ACT001 dose levels were 100 mg BID, 200 mg BID, 400 mg BID and 600 mg BID. Study treatment was well tolerated and no dose-limiting toxicities have occurred up to ten months of treatment. Thus, both this phase 1 clinical trials result in human patients and our findings in mice suggest that long-term administration of DMAMCL is well-tolerated and safe. Therefore, DMAMCL may have a potential to be a promising aging intervention agent. Based on all results we obtained from three doses of DMAMCL intervention in mice, we suggest low to median dose of DMAMCL (around 15 to 20 mg/kg/EOD) to be used for future more comprehensive aging intervention study in mice.

Our study had several limitations. (1) We examined only a single inbred mouse strain and gender (i.e., male C57BL/6 mice). Whether DMAMCL has effect on aging in other strains and/or in female needs to be investigated in the future. (2) We began DMAMCL treatment at 12 months of age (middle aged mice) and ended at 27 months of age. We did not examine additional intermediate or older cohorts. However, from survival curves we noticed that the survival rate of control and DMAMCL-treated male mice started to separate at late intervention period (Fig. 1D, and Fig. S2A-C). There were more mice survived in all three doses of DMAMCL-fed mice than in vehicle-treated mice at the end of study. This result implies that the improvement of healthy aging by DMAMCL treatment may be more obvious and effective when starting aging intervention at old age in mice, namely around 20 to 22 months of age. This aging intervention strategy at old age may have more translational medicinal implication. The hypothesis needs to be tested in the future. (3) Due to limited sample sizes in each group, we ended aging intervention at 27 months of age. Therefore, we did not obtain lifespan extension effect of DMAMCL treatment on mice. To assess whether long-term administration of DMAMCL can extend lifespan in mice either in middle age or old age intervention, larger sample sizes are needed to determine the lifespan extension effect of DMAMCL in the future.

In conclusion, in this study we show for the first time that long-term administration of DMAMCL may ameliorate or has little effect on some age-associated physiological decline in mice. Meanwhile, we demonstrate that the chronic DMAMCL treatment is well tolerated and safe in mice. Considering DMAMCL is undergoing clinical trials, our ﬁndings from this long-term administration study may provide some clues to further investigate whether DMAMCL can be an effective aging intervention compound.

## MATERIALS AND METHODS

### Animals

Ninety-two male C57BL/6 mice at 7 months of age were purchased from Vital River Laboratory Animal Technology Company LTD (Beijing, China). The animals were individually housed in stainless steel cages in the Department of Laboratory Animal Science, Peking University Health Science Center and were maintained on a commercial diet with tap water ad libitum until 54 weeks of age. The room was maintained at 22 ± 2°C with 55 ± 5% humidity and a 12-h light/dark cycle. At 54 weeks of age, the animals were randomly divided into four groups: (A) control group (n = 23), (B) low dosage group of ACT001 (10 mg/kg/EOD; n = 23), (C) medium dosage group of ACT001 (25 mg/kg/EOD; n = 23), and (D) high dosage group of ACT001 (50 mg/kg/EOD; n = 23). DMAMCL was generously provided by Accendatech Co., Ltd (Tianjin, China) and obtained as a white powder with a molecular weight of 409.47, general formula C_17_H_27_NO_3_·C_4_H_4_O_4_, and 99.12% purity. The powder was tightly sealed to prevent from humidity and stored in the dark at 4°C and was dissolved in sterile water when used. In order to ensure the accuracy of the dosages, the mice were given DMAMCL every other day by intragastric administration. The control group was given an equal amount of sterile water. Body weight was measured biweekly for the duration of the study. Throughout their lifespan, the mice were observed once daily, and the precise date of death of each mouse was recorded. Survival curves were plotted using the Kaplan–Meier method, which includes all available animals at each time point. All research protocols were approved by the Animal Welfare Committee of Peking University Health Science Center (LA2017173, approved in February 28, 2017).

### Rotarod

An accelerating rotarod was used to measure motor coordination and balance. For the rotarod test, mice were given a habituation trial on day 1 where they were placed on the rotarod at a constant speed (4 rpm) and had to remain on the rotarod for 1 min. The following day, mice were placed on the rotarod, and the rod rotations were subsequently accelerated from 4 to 40 rpm during the 5-minute trial period. Trials were terminated when each animal fell off the rod for total three times. Each animal were given 3 trials, with intertrial intervals of 30 minutes. Latencies to fall data (s) were analyzed across groups and presented as the average of the three trials for each group (n = 8-12 mice per group).

### Open field test

Exploratory locomotor activity was assessed by an open field test. The apparatus consisted of a gray box that was open at the top and the floor was divided into the central area and the out ring. Each mouse was placed gently on the field and movement was recorded for 5 minutes. The measurements included total distance and duration of movement in the central area. After each mouse had been tested, the box was thoroughly cleaned to remove odor cues (n = 13-14 per group).

### Treadmill

For the treadmill test, mice were required to exercise on the treadmill until exhaustion. The treadmill was horizontal (0° incline) and mice ran in groups of six. Animals were habituated at a constant speed of 4 m min^-1^ for 5 min at day 1. The following day, each mouse was given a trial starting at 7 m min^-1^ for 0-3 min, 12 m min^-1^ for 3-7 min, 15 m min^-1^ for 7-25 min and 19 m min^-1^ for 25 min. Mouse was considered as exhaustion when animal got three electric shocks within 10 sec or could not run out of the electric shock zone. Total distance and time ran in the treadmill test until exhaustion were recorded (n = 9-10 per group).

### Elevated plus maze

The elevated plus maze (EPM) consisted of two opposite open arms (50 ×10 cm) and two enclosed arms (50 ×10 cm, surrounded by a 40 cm high black wall) elevated 75 cm from the floor. Individual trials lasted 5 min for each. An entry into an arm was deemed to have occurred when all four paws and the base of tail were inside the arm. At the beginning of each trial, animals were placed at the center of the maze, facing an open arm. The maze was cleaned with 75% (v/v) ethanol solution after each trial. The number of entries and the time spent in open arms were measured in addition to the number of entries and the time spent in enclosed arms. Open-arm exploration was measured by normalizing open-arm time (OT) to total time (time spent in open arm + time spent in enclosed arm). In this paradigm, anxiety is measured as a function of decreased open-arm exploration (n = 14 per group).

### Morris water maze

Each mouse was trained to find a platform visible underneath the water surface in a constant location of the pool for 4 times at day 0. Then, each mouse received 4 daily training trials in the hidden version of the Morris water maze for 5 consecutive days. Training trials were completed when mice climbed on the escape platform or when 1 minute had elapsed, whichever came first. To evaluate the accuracy with which the animals had learned the position of the escape platform, we performed a probe trial at day 6 after completion of training. We determined the first time to find platform location at day 6 (first time-D6) and the average speed to find the probe at day 6 (D6-probe_average speed). Additionally, we determined the time that mice spent searching in the target quadrant (TQ, which previously contained the escape platform) or the other quadrants (OQ) during the probe trial (n = 12 per group).

### Bone structure and body composition analysis

BMD (bone mineral density) and body composition (lean weight and fat weight) were determined using an UltraFocus DXA Bone Densitometer (Faxitron Bioptics, USA). Fat percent and lean/fat ratio were calculated (n = 10 per group).

### Echocardiography

Cardiac function was assessed using an echocardiography with the Vevo 770TM Imaging System (Visual Sonics Inc., Toronto, Canada) equipped with a 30-MHz microprobe under anesthesia with 1.5% isoflurane allowing spontaneous breathing. Left ventricular parasternal short- and long-axis views were obtained in B-mode imaging and left ventricular parasternal short-axis views were obtained in M-mode imaging at the papillary muscle level. Left ventricular internal diameter in diastole (LVIDD), left ventricular internal diameter in systole (LVIDS), left ventricular posterior wall in diastole (LVPWD), and left ventricular posterior wall in systole (LVPWS) were determined using short-axis M-mode images derived from three consecutive beats. Fractional shortening (FS), ejection fraction (EF), and E/e’ ratio was calculated according to the standard methods (n = 6-8 per group).

### Blood collection

Mice’s eye orbit was punctured with non-heparinized glass capillaries (1.0 mm in diameter) to collect blood samples. Blood samples were collected in heparinized sample tubes. Each tube was immediately inverted ﬁve times to achieve a homogeneous distribution of the anticoagulant. Heparin-coated tubes were stored at room temperature for 1-2 h. Afterwards, cells and plasma were separated by a centrifugation step (10 min, 5000 × g, 8 °C). Plasma was used for the clinical chemistry assessment and Luminex screening assay. The cell pellet was used for FACS-based analysis of peripheral blood leukocytes (PBLs).

### Hematology

A measure of 20 µl EDTA blood was diluted 1:5 in 100 μl of Sysmex Cell-Pack buffer prefilled tubes and was used to determine complete blood cell counts using HEMAVET 950 Hematology Analyzer (n = 10 per group).

### Clinical chemistry

Serum metabolites were measured using a Mindray BS-180 Automatic Biochemical Analyzer (Mindray, Shenzhen, China) (n = 7-10 per group).

### Luminex screening assay to detect blood levels of inflammatory cytokines

Blood levels of IL-6, IL-1α, IL-1β, TNF-a, IFN-γ, CXCL2, and GM-CSF were measured using a mouse magnetic Luminex screening assay with commercial kits (RnD, Cat# LXSAHM-07, R&D Systems Europe, Abingdon, UK), according to the manufacturers’ instructions (n = 10 per group).

### FACS-based analysis of peripheral blood leukocytes (PBLs)

PBLs were proﬁled from 100 µl whole blood per mouse. Each whole-blood sample was incubated with the antibody mix at room temperature for 20 min. Next, erythrocytes were lysed with erythrolysin for 3 min (BD Pharm Lyse Lysing Buffer, Cat: 555899). Cells were then centrifuged at 300g for 5 min, and cell pellets were washed twice with PBS buffer (PH = 7.2-7.4) and followed by centrifugation. Finally, cells were suspended in PBS buffer and were analyzed on a ten-color ﬂow cytometer (Gallios, Beckman Coulter). The acquisition threshold was set on the CD45-channel. A total number of 80,000-140,000 leukocytes per sample was examined. A software-based analysis (AS49313 Software Version: Gallios 1.1) was used to quantify individual PBL frequencies (n=7-10 per group).

The following main leukocyte populations were examined: T cells (CD45.2^+^CD3^+^), Ly6C^+^ T cells (CD45.2^+^CD3^+^ Ly6C^+^), B cells (CD45.2^+^CD19^+^), granulocytes (CD45.2^+^Ly6G^+^), NK cells (CD45.2^+^NK1.1^+^), Ly6C^+^ NK cells (CD45.2^+^NK1.1^+^ Ly6C^+^), Ly6C^+^ monocytes (non-lymphocytes, CD14^+^ Ly6C^+^).

### OGTT

Mice were fasted for 6 h and received an oral dose of 2 g/kg of glucose (Sigma-Aldrich, St. Louis, MO) by gavage. At baseline and 15, 30, 60, 90 and 120 min after glucose administration, blood glucose levels were determined by tail venipuncture using an ONETOUCH UltraEasy Glucose Meter (n = 10 per group).

### Isolation of total RNA and RT-PCR

Total RNA was extracted from fat tissue using TRIzol reagent (Invitrogen, Carlsbad, CA, USA) following the manufacturer's instructions. Reverse transcription was performed using RevertAid First Strand cDNA Synthesis Kit (Thermo Scientific). Real-time PCR analysis was performed using SYBR Select Master Mix (Life technologies) in conjunction with an ABI Prism 7500 Sequence Detection System with the expression of β-actin as the internal control. The data were analyzed using the ΔΔCT method. The primers for TNF-α and β-actin were described as below.

TNF-α:

F: 5’-GAAAAGCAAGCAGCCAACCA-3’,

R: 5’-CGGATCATGCTTTCTGTGCTC-3’;

β-actin:

F: 5’-TCCTCCTGAGCGCAAGTACTCT-3’,

R: 5’-GCTCAGTAACAGTCCGCCTAGAA-3’.

### Western blot analysis

The protein expressions of p65, p-p65, IκBα, Nrf2, and HO-1 were determined by Western blot analysis. Antibodies against p65, p-p65 and IκBα were purchased from Cell Signaling (Beverly, MA, USA), antibody against Nrf2 was purchased from Ruiying Biological, and antibodies against GAPDH, β-actin and β-tubulin were purchased from Bioworld. Tissues were lysed in RIPA buffer (Applygen Technologies) with phosphatase inhibitor tablet (Roche Diagnostics) and protease inhibitor (Cocktails, AMRESCO). Lysates were then centrifuged for 15 min at 12,000 × g at 4 °C and supernatants were collected and protein concentrations were determined by BCA Protein Assay Reagent (Pierce). Cell lysates (15-30 μg) were subjected to 10% SDS-polyacrylamide gel electrophoresis (SDS/PAGE) and transferred to nitrocellulose membranes (Millipore). For western blotting analysis, membranes were incubated with primary antibodies for overnight at 4ºC followed by incubation with a secondary antibody for 1h at r.t. Then the signals were detected by enhanced chemiluminescence according to the manufacturer’s recommendation.

### Statistical analysis

In all experiments, data were presented as the mean ± SEM. Unless otherwise stated, Student’s t-test was used to analyze statistical differences between groups. Statistical analyses were carried out using GraphPad (version 6.01). A two-tailed *P*-value of less than 0.05 was considered significant. *P < 0.05, ** *P* < 0.01.

## SUPPLEMENTARY MATERIAL

Supplementary Figures
