# Supplementary material for: Long-term every-other-day administration of DMAMCL has little effect on aging and age-associated physiological decline in mice
Source: Aging (Albany NY). 2019 May 2;11(9):2583–609. doi: 10.18632/aging.101932 (PMC6535057; doi:10.18632/aging.101932)
Supplement: Supplementary Figures [file aging-11-101932-s001.pdf]

SUPPLEMENTARY MATERIAL

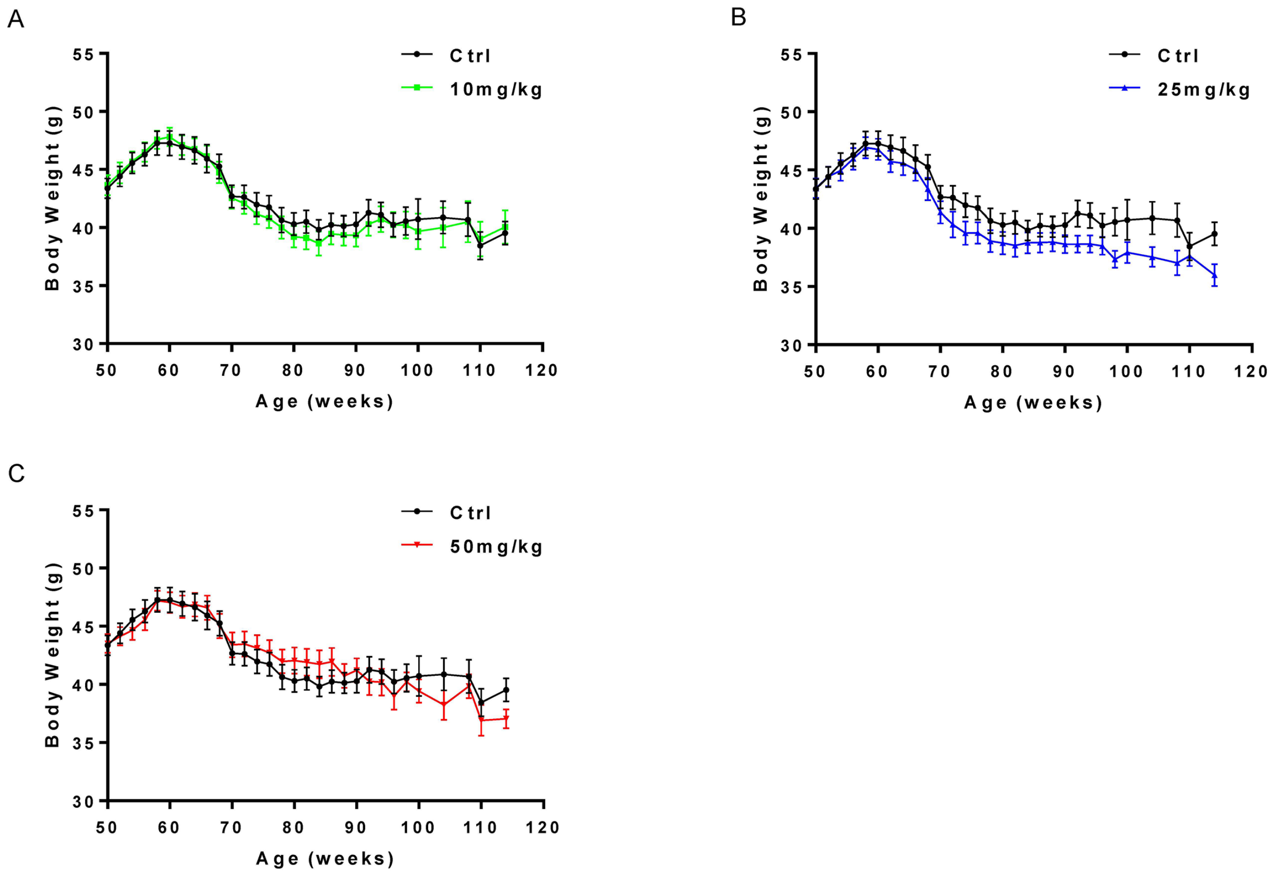

Figure S1. Effects of DMAMCL on body weight. (**A**) Body weight of control and 10 mg/kg/EOD-fed mice. (**B**) Body weight of control and 25 mg/kg/EOD-fed mice. (C) Body weight of control and 50 mg/kg/EOD-fed mice.

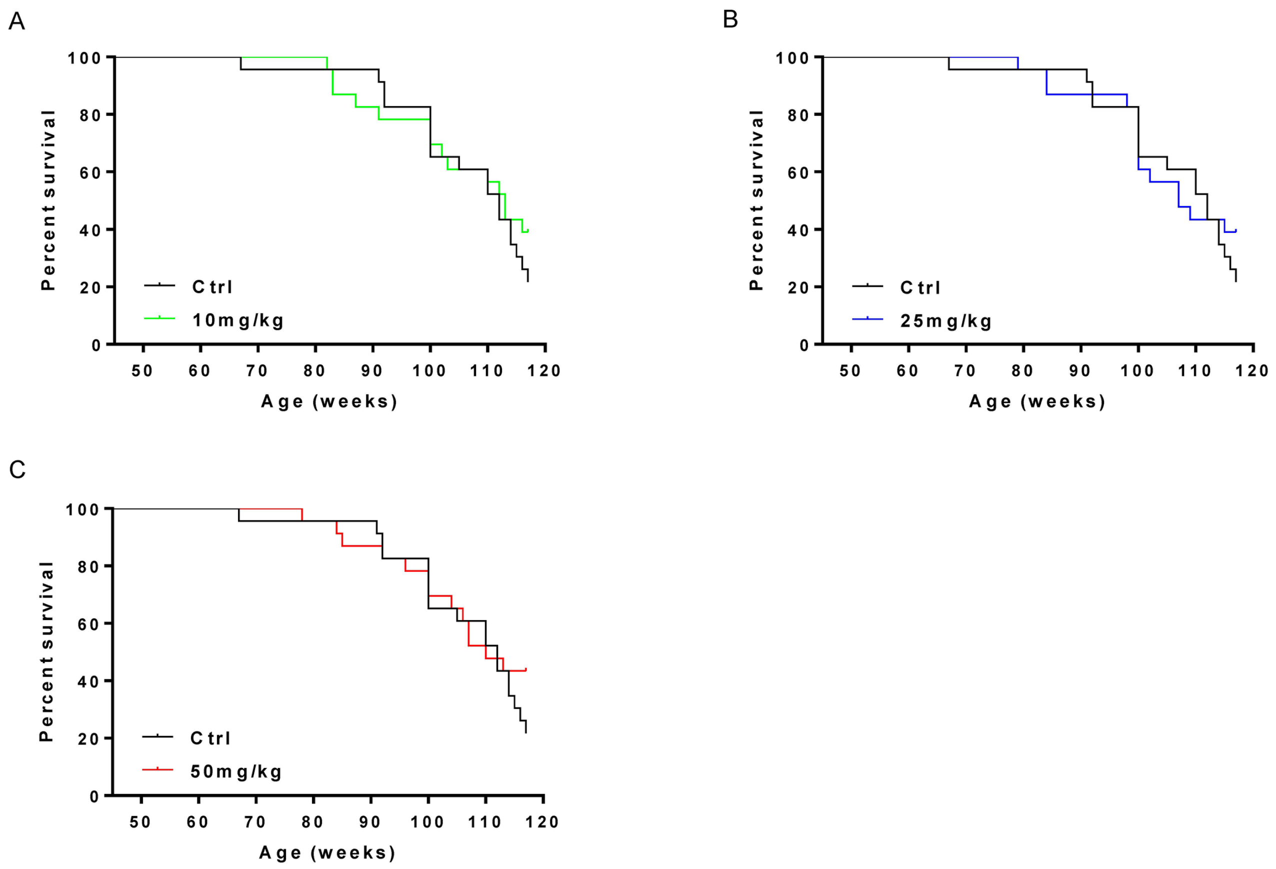

Figure S2. Effects of DMAMCL on survival. Kaplan-Meier survival curve for mice treated with low (**A**) (log-rank test: P = 0.4143), median (**B**) (log-rank test: P = 0.4962) and high (**C**) (log-rank test: P = 0.3514) doses of DMAMCL.

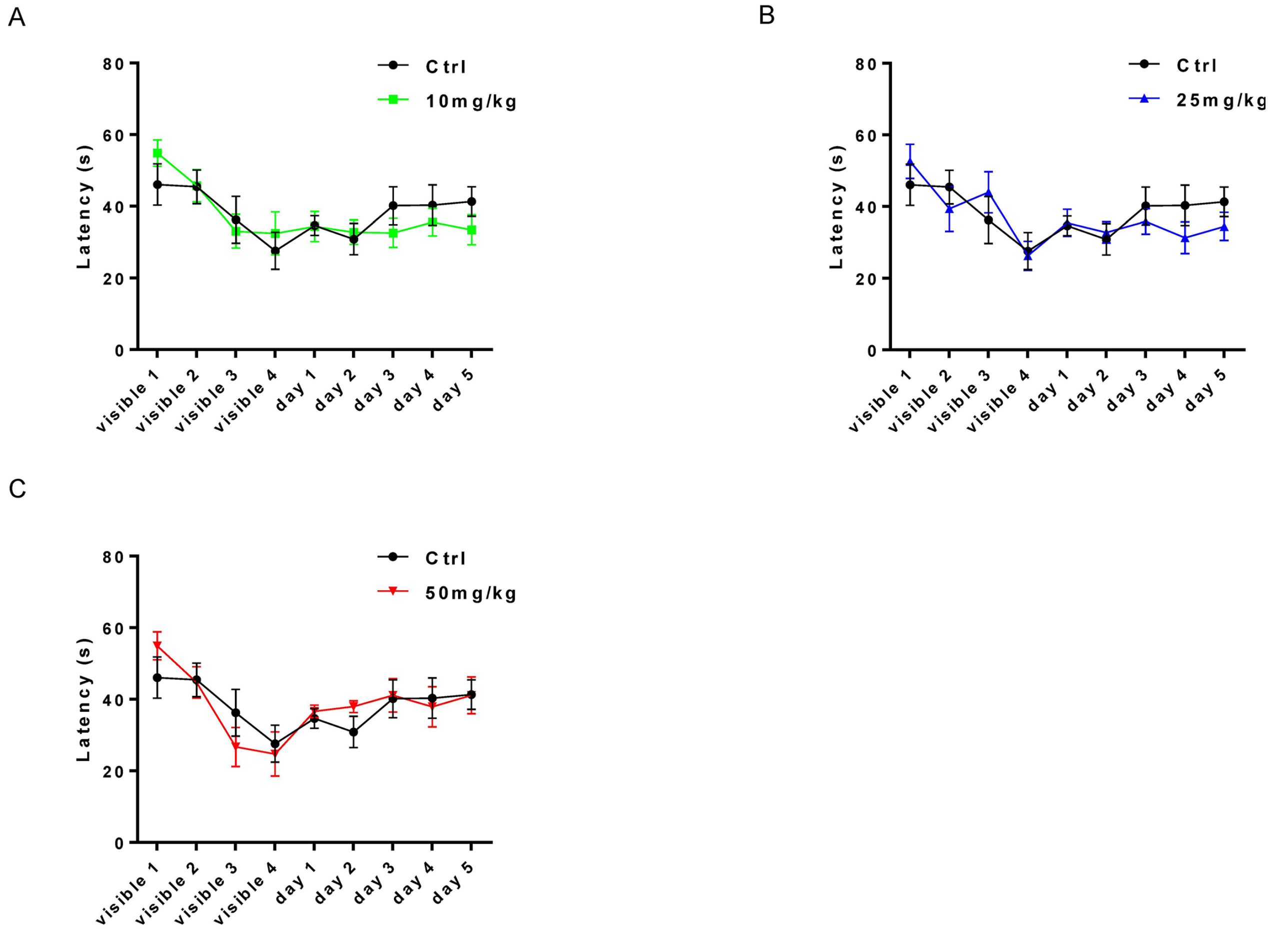

Figure S3. Latency (the time to find platform) of mice treated with low (**A**) (two-way repeated-measures ANOVA: treatment x time $F(8,160) = 0.8227$, $P = 0.5837$; time $F(8,160) = 4.484$, $P < 0.0001$; treatment $F(1,20) = 0.07017$, $P = 0.7938$), median (**B**) (two-way repeated-measures ANOVA: treatment x time $F(8,160) = 0.9149$, $P = 0.5057$; time $F(8,160) = 4.244$, $P < 0.0001$; treatment $F(1,20) = 0.1172$, $P = 0.7356$), and high (**C**) (two-way repeated-measures ANOVA: treatment x time $F(8,160) = 0.8062$, $P = 0.5980$; time $F(8,160) = 5.745$, $P < 0.0001$; treatment $F(1,20) = 0.009139$, $P = 0.9248$) doses of DMAMCL in the Morris water maze assay.

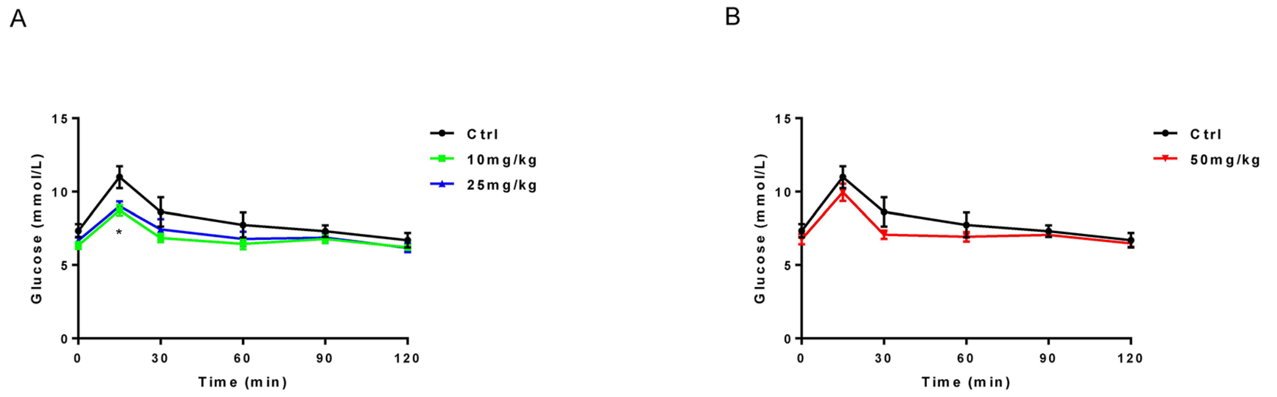

Figure S4. Assessment of glucose homeostasis. (**A**) OGTT curves of control and low and median doses of DMAMCL-treated mice (n =10). (**B**) OGTT curves of control and high dose of DMAMCL-treated mice.

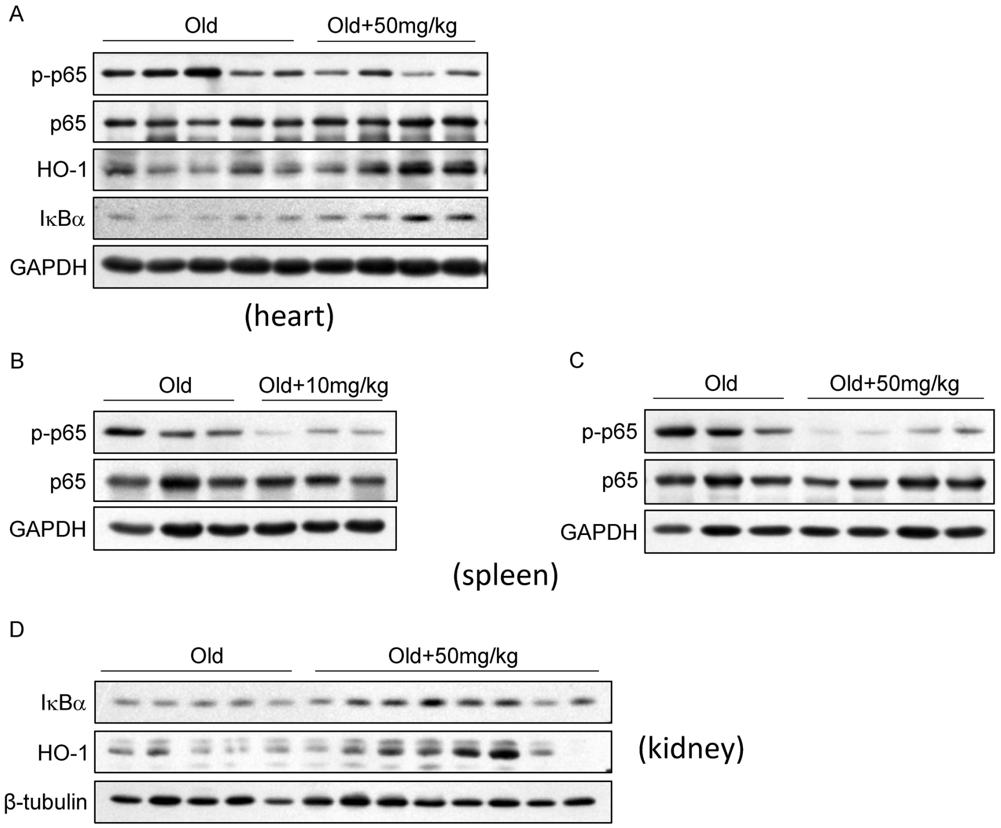

Figure S5. Effects of DMAMCL on protein expression in multiple tissues in old mice. The total protein was isolated from the heart, spleen and kidney tissues and protein expression were examined by Western blot (n=3-8). (**A**) The effect of high dose DMAMCL treatment on the p-p65, p65, HO-1 and IκBα protein expression in heart. (B and C) The expression levels of p-p65 and p65 in spleen were examined in mice treated with low (**B**) and high (**C**) doses of DMAMCL. (**D**) The effect of high dose DMAMCL treatment on the IκBα and HO-1 protein expression in kidney.
